# Untangling the Rhizosphere Bacterial Community Composition and Response of Soil Physiochemical Properties to Different Nitrogen Applications in Sugarcane Field

**DOI:** 10.3389/fmicb.2022.856078

**Published:** 2022-03-14

**Authors:** Abdullah Khan, Hongtao Jiang, Junyao Bu, Muhammad Adnan, Syeda Wajeeha Gillani, Muhammad Azhar Hussain, Muqing Zhang

**Affiliations:** ^1^Guangxi Key Laboratory of Sugarcane Biology, State Key Laboratory for Conservation and Utilization of Subtropical Agro-Bioresources, Guangxi University, Nanning, China; ^2^Alfa Diagnostic Services, Faisalabad, Pakistan

**Keywords:** sugarcane, rhizosphere, nitrogen, soil enzymes, bacterial diversity, yield

## Abstract

Minimizing the use of chemical fertilizers and investigating an appropriate ecofriendly level of nitrogen fertilizer is the key to sustainable agriculture. Sugarcane is the main cash crop of China, especially in the Guangxi region. Information regarding the effect of different nitrogen levels on sugarcane rhizosphere microbiota is still limited. In this study, we evaluated the effect of four different levels of nitrogen fertilizers on rhizosphere bacterial composition using high throughput sequencing, along with soil physiochemical properties, sugarcane agronomic and yield performance. The four treatment combinations were CK (no fertilizers), L (Low, 100 kg ha^–1^), M (Medium, 150 kg ha^–1^), and H (High, 200 kg ha^–1^). The results showed that M nitrogen application significantly altered the rhizosphere bacterial community, soil properties, and sugarcane yield. The richness and evenness of the bacterial community were higher in M treatment than CK. In M treatment important bacterial phyla Acidobacteria and Proteobacteria increased by 47 and 71%, respectively; and at genus level, *Acidothermus* and *Bradyrhizobium* increased by 77.2 and 30.3%, respectively, compared to CK. Principal component analysis (PCA) and cluster analysis further confirmed the level of differences among the treatments. The PCA analysis explained 80% of the total variation among the treatments. Spearmen correlation heatmap showed that environmental factors such as pH, AP (available phosphorous), AK (available potassium), and SCAT (soil catalase) were the key factors impacting sugarcane rhizosphere microbiome composition. The H and L nitrogen application alter the bacterial community and sugarcane performance but the M nitrogen application appears to be ecofriendly, productive, and an appropriate nitrogen application rate that could be further used in the Guangxi region.

## Introduction

Sugarcane (*Saccharum officinarum* L.) is an imperative sugar and energy crop worldwide. It is a long-duration crop that grows quite tall and absorbs more nutrients, requiring more fertilizers, irrigation, and a healthy soil fertility balance (Li et al., 2020). China ranks third in sugar production worldwide, having 90% of sugar production from southern and southwest regions. In these regions particularly, the Guangxi region accounts for 65% of the total sugarcane production in China for the last three decades (Yang et al., 2021). However, continuous monoculture (Tayyab et al., 2021) and excessive nitrogen fertilizer usage (Gu et al., 2021), resulting in low cane yield is a still a point of concern in China. Growing demand for sugarcane and biofuel drives the worldwide sugarcane industry to increase production. The sugarcane industry is experiencing diminishing yields despite significant N fertilizers input and intensive agronomy (Yeoh et al., 2016). Sugarcane crops consume around half of the fertilizer applied globally (Robinson et al., 2011), which is similarly inefficient as other agricultural crops (Chen et al., 2008).

N metabolizing microorganisms obtain energy from N fertilizer by urea hydrolysis, nitrification, and denitrification, producing nitrates and other forms of N (Wrage et al., 2001; Ali et al., 2021). Soil acidification and soil organic matter loss are two further issues associated to the usage of synthetic N fertilizers (Guo et al., 2010; Muhammad et al., 2018; Ali et al., 2020). With N derived by diazotrophic endophytic bacteria such as acetobacter (Cavalcante and Dobereiner, 1988; Boddey et al., 2001) sugarcane does not require intensive N fertilizer. Conversely, higher concentrations of N inhibit the growth of diazotrophic bacteria (Fuentes-Ramirez et al., 1999). Chemical fertilizers were overused to achieve maximum yield by farmers in the Guangxi region, where the application of N fertilizers varied from 600 to 800 kg ha^–1^ compared to other sugarcane-producing countries (Li and Yang, 2015).

External environmental conditions have a significant impact on sugarcane fertilizer application, with evident regional variability. In China, research regarding optimum fertilizer application for sugarcane is still in the early stage, and no clear conclusion regarding an appropriate and productive rate has been achieved yet (Niu et al., 2021). Plant-associated microbial diversity (rhizospheric and endophytic microbes) directly impacts crop productivity. Biogeochemical soil nutrient cycling, biological nitrogen fixation, breakdown of organic material, plant growth stimulation, and resistance to disease and abiotic stresses are all controlled by these microbes (Arif et al., 2020; Muhammad et al., 2021b; Santos et al., 2021). Microbes in the rhizosphere can affect the rhizophagy cycle in roots and ultimately modify hormonal interaction in other plant parts (White et al., 2019). Because of their functional contributions and connection with microorganisms called keystones, the composition of microbial structures in plants and agricultural soils has been a focus of research (Beltran-Garcia et al., 2021). Soil fertility is judged by many factors, including soil organic matter which is an important one. The activity of microorganisms is believed to be increasing with an increase in total soil organic matter content that can accurately reflect changes in soil nitrogen distribution (Gyaneshwar et al., 2002; Muhammad et al., 2021a). Microbial activity is greatly affected by the use of chemical fertilizer. Application of chemical fertilizers can greatly improve the soil microbial environment, resulting in a varying amount of soil carbon stowage, altering the functional microbial assemblages, and ultimately affecting carbon sink in the terrestrial ecosystems. However, the understanding of microbial diversity in the sugarcane field using chemical fertilizer, especially nitrogen fertilizer, is limited.

Organic manure can increase crop yields (Maltas et al., 2013; Van der Bom et al., 2018) and effect the relative abundance of soil microbes, however, this has been due to the long-term organic manure application and an increased level of SOM (Soil organic matter) (Li et al., 2015). Studies concerning the impact of only organic fertilizer application or in combination with inorganic fertilizer have shown significant results and changes in the microbial community populations. However, the short-term impact of chemical fertilizer on soil microbial communities in sugarcane is still on the move. Therefore, in view of this, the present research was conducted to investigate (1) whether different rates of nitrogen fertilizer have a simultaneous effect on the sugarcane rhizosphere microbial community structure (2) what is the appropriate/optimum rate of nitrogen fertilizer application for sugarcane in southern China, and (3) how it impacts sugarcane performance and yield.

## Materials and Methods

### Experimental Location

This field experiment was conducted in the Forage and Breeding ground in Chongzou, China (22°38′06″N,107°54′15″E) of Guangxi University in 2021. The field station is a major sugarcane growing area in the Guangxi Autonomous region. The mean annual temperature was 24.1°C. The highest temperature recorded during the past year was 37°C, while the lowest was 4°C. The annual precipitation recorded in the region was 1182.2 mm. The site is dry and windy in winter and rainy and humid in summer.

### Experimental Design and Treatments

The experiment was conducted in a randomized complete block design with three replicates. The tested sugarcane genotype ZZ-13, an offspring of HOCP01-157 × CP14-0969, was released by Guangxi University. The genotype is high yielding and high sugar content variety. The experiment consists of 12 blocks each having an area of 60 m^2^ with a row length of 15 m. The distance between two lines was kept 2 m while plant to plant distance was 30 cm. For chemical fertilizer (NPK), urea (46% nitrogen), single superphosphate (20% phosphorous) and Muriate of potash (60% potassium) were taken as a source and applied in two splits. At the germination stage 60% of the fertilizers were applied to the field while, 40% at the Grand growth stage. The experiment consisted of 4 treatments: CK (no fertilizers), L (Low, 100 N kg ha^–1^), M (Medium, 150 N kg ha^–1^) and H (High, 200 N kg ha^–1^). As basal fertilizer phosphorous and potassium were likewise applied in 4 different treatments. All other agronomic practices were kept uniform for all the treatments throughout the experiment.

### Samples Collection and Estimation of Theoretical Yield

Morphological data including plant height, stem diameter, number of nodes and internode length was recorded in the end of December followed by collection of rhizosphere soil samples. Data was recorded on 30 plants per replicate and average value was computed. A meter rod was used to calculate plant height. The number of nodes in each plant was counted and the mean value was recorded. Stem diameter and internode length was recorded for each treatment from the plant top to every tenth internode using vernier caliper and inch tape, respectively. Soil particles adhered to the roots were collected as rhizosphere and stored in three parts for soil analysis after being sieved by 2 mm mesh. Sucrose content in each treatment was recorded with the help of a portable refractometer ATAGo Pocket PAL-1 (Atago Co., Ltd., Tokyo, Japan). The single cane weight (kg) and theoretical cane production was calculated with the following equation (Pang et al., 2021; Khan et al., 2022).


(A)⁢S⁢i⁢n⁢g⁢l⁢e⁢s⁢t⁢a⁢l⁢k⁢w⁢e⁢i⁢g⁢h⁢t⁢(k⁢g)=

$$\frac{{\left(S\,t\,a\,l\,k\,d\,i\,a\,m\,e\,t\,e\,r\,\left(c\,m\right)\right)}^{2}\times \left(s\,t\,a\,l\,k\,h\,e\,i\,g\,h\,t\,\left(c\,m\right)-30\right)\times 1\,\left(\frac{g}{c\,m^{3}}\right)\times 0.7854}{1000}$$

$$\left(B\right)\,C\,a\,n\,e\,y\,i\,e\,l\,d\,\left(\frac{t}{h\,a^{-1}}\right)=$$

$$\frac{S\,i\,n\,g\,l\,e\,s\,t\,a\,l\,k\,w\,e\,i\,g\,h\,t\,\left(k\,g\right)\times s\,t\,a\,l\,k\,n\,u\,m\,b\,e\,r\,s\,\left(n\,o\,h\,a^{-1}\right)}{1000}$$

### Soil Physiochemical Properties

One part of the stored soil sample was used for measuring soil enzymatic activity such as urease (S-UE), catalase (S-CAT) and acid phosphatase (S-ACP) through soil enzyme kits, following manufacturer’s instructions from Solarbio Science and Technology Co. (Biejing, China). The second part of the soil was used for analysis of chemical properties, including SOC (soil organic carbon), AN (available nitrogen), AP (available phosphorous) and AK (available potassium). For SOC, content soil was oxidized with K_2_Cr_2_O_7_.H_2_SO_4_ and titrated with FeSO_4_ (Cambardella et al., 2001). Available nitrogen, phosphorous, and potassium were measured according to the method described by Murphy and Riley (1962), Knudsen et al. (1983), and Dorich and Nelson (1984), respectively.

### DNA Extraction and PCR Amplification

Rhizospheric soil DNA from each sample was extracted using FASTDNA™ Spin Kit for soil (CO. MP Biomedicals, United States) following manufacturer’s instructions. The quantity of each extracted DNA was measured with NanoDrop 2000 (Thermo Fisher Scientific, Wilmington, United States). The bacterial v5-v7 region was amplified using 799F (forward primer, 5-AACMGGATTAGATACCCKG-3) and 1193R (reverse primer 5-ACGTCATCCCCACCTTCC-3) (Khan et al., 2021) and DNA samples as amplification template. The PCR experiments were carried out in a 25-μL reaction mixture including 20 ng DNA template, 0.5-μL dNTP, 10-μL of KOD (kodakaraensis) polymerase Buffer, 0.25-μL DNA polymerase, 5-μL High GC enhancer, and 1.0-μL of each primer (Pang et al., 2019). The PCR thermal conditions were as follows: the first denaturation was carried out for 5 min at 98°C, followed by 25 cycles at 94°C for 30 s, 52°C for 30 s (annealing), 72°C for 30 s (extension), and 72°C for 10 min (final elongation). A Bio-Rad S1000 thermocycler was used to perform PCR amplification (Bio-Rad Laboratories, CA, United States). After the reaction, products were mixed evenly, and the target bands were spotted using a 2% agarose gel electrophoresis. QIAamp DNA Micro Kit was used to retrieve the intended bands (Qiagen, Valencia, CA, United States). Following that, using the Illumina TruSeq DNA sample preparation kit, DNA libraries were created (Illumina, San Diego, CA, United States). The Illumina HiSeq2500 platform was used to perform high-throughput sequencing of 16S rRNA, resulting in 250 bp paired-end reads, by the Gene *Denovo* Biotechnology Co., Ltd. (Guangzhou, China). Finally, raw metagenomics and datasets were deposited in the NCBI Sequence Read Archive (SRA) database with a BioProject ID: PRJNA798064.

### Data Analysis

Using FLASH software, raw tag sequences were checked for quality and combined into clean reads. To acquire valid sequences for each sample, the clean reads were assigned to the relevant sample. The downstream analysis was carried out using the QIIME (Quantitative Insights into Microbial Ecology v.1.9.0) program. By using the pairend data as an input file in QIIME software, the operational taxonomic unit (OTU) was allocated to representative sequences. OTUs were identified at a 97% similarity criterion using the UCLUST algorithm and the Greengene database as a reference database (Flynn et al., 2015). Within each sample, each OTU sequence represented the taxonomy, including phylum class, order, family, genus, and species. The generated OTU table was then analyzed using Microbiome Analyst (Chong et al., 2020). The input data was rarefied to the smallest library size possible using the default functions for total sum normalization. Filtering of low variance was set at 20% having the interquartile range, and the sequences were filtered at a minimum of 4 with a 20% prevalence in the sample. For each sample, each distinct taxa were computed using the relative abundance. The diversity indices Chao1, ACE, Simpson, and Shannon were calculated for each sample, and a rarefaction curve was constructed using Mothur (v.121.1). Alpha diversity was used to characterize the diversity inside a single sample. The R function was used to perform a beta diversity analysis to see if there were any differences or similarities between the treatments.

## Results

### Cane Morphological Parameters and Yield Index

**FIGURE 1:**
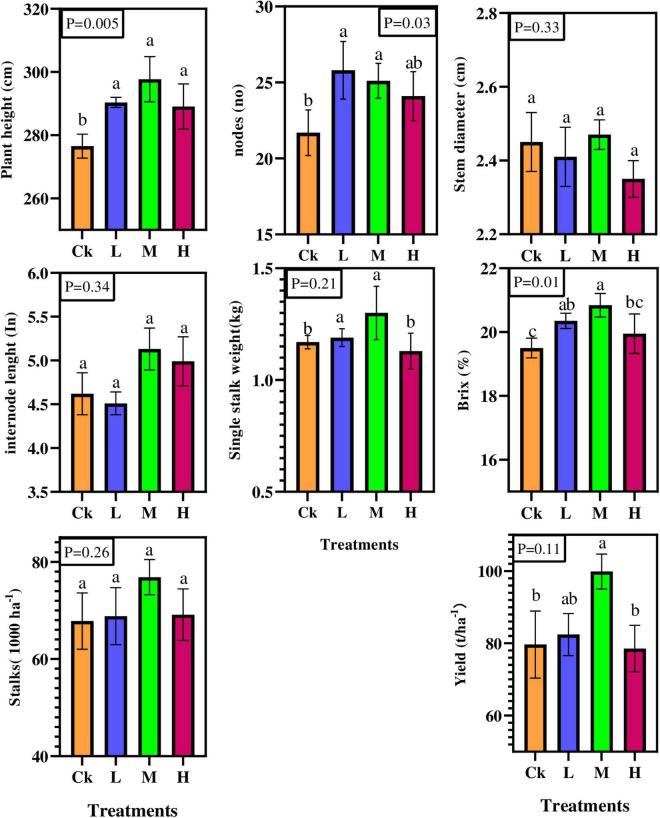
Sugarcane morphological and yield characteristics under four different nitrogen fertilizer applications. CK; no fertilizers, L = (Low, 100 kg ha^–1^) M = (Medium, 150 kg ha^–1^) H = (High, 200 kg ha^–1^).

Results showed that nitrogen, phosphorous and potassium fertilizers significantly affected the number of nodes, plant height, and sugar content (*p* < 0.05; Figure 1). Compared to Ck, plant height and sugar content were statistically and significantly (*P* < 0.05) increased in M treatment by 7.66 and 6%, respectively, except the number of nodes was statistically significant in L treatment. However, variations were observed in internodes length, stem diameter, single stalk weight, available stalks number, and cane yield, but their effect was statistically insignificant. Furthermore, compared to Ck, the sugarcane yield per hectare varied from 4 to 25% under different fertilizer rates. Overall, the results showed that sugarcane morphological attributes were improved by different nitrogen applications compared to control treatment.

### Soil Physiochemical and Enzymatic Properties

Changes in soil physiochemical properties and enzymes activity in response to different nitrogen, phosphorous and potassium application is shown in Table 1. Among the treatments, soil pH value increased by 29 and 10% in H and M treatment, respectively, as compared to Ck. Similarly, SOC, AN, AP and AK were enhanced by 35.8, 25.9, 13.4, and 9.54% in M treatment, respectively, as compared to Ck. Soil enzymes activity including SCAT, SUE and SACP were also improved in M treatment by 90.8, 67.75, and 43.05% over Ck. SCAT was found to be altered significantly (*P* < 0.05) compared to SUE and SACP. In general, control treatment resulted in the lowest values of soil pH (4.13), SOC (17.90 g kg^–1^), AN (92.6 mg kg^–1^), AP (43.62 mg kg^–1^), and AK (107.04 mg kg^–1^) and less activity of soil enzymes (SCAT, SUE and SACP) as compared the fertilizer applied treatments.

**TABLE 1:** Effect of different treatments on soil nutrients and enzymatic activity.

Treatments	PH value	SOC (g.kg^–1^)	AN (mg.kg^–1^)	AP (mg.kg^–1^)	AK (mg.kg^–1^)	SCAT (U/g)	SUE(U/g)	SACP (μmol/d/g)
**CK**	4.13 ± 0.05c	17.90 ± 1.58b	92.6 ± 1.70d	43.62 ± 0.88c	107.04 ± 1.4*bc*	13.20 ± 2.94b	189.54 ± 21.15a	36.36 ± 3.11a
**L**	4.54 ± 0.09b	21.83 ± 2.47*ab*	103.7 ± 0.90c	47.15 ± 1.07b	110.51 ± 1.91b	18.03 ± 3.53*ab*	232.45 ± 16.32a	46.77 ± 3.80a
**M**	4.58 ± 0.05b	24.32 ± 1.56a	116.3 ± 1.40a	49.50 ± 0.48a	117.26 ± 1.40a	25.20 ± 3.17a	317.96 ± 10.8a	52.01 ± 3.01a
**H**	5.37 ± 0.08a	24.38 ± 2.74a	113.17 ± 0.69b	46.91 ± 1.44b	102.90 ± 1.46c	18.25 ± 3.30*ab*	265.94 ± 14.83a	42.29 ± 2.84a
**F**	137.72	5.10	1225.73	24.08	19.03	4.90	1.54	1.84
**P**	<0.001	<0.05	<0.001	<0.05	<0.01	<0.05	= 0.29	= 0.24

SOC, soil organic carbon; AN, available nitrogen; AP, available phosphorous; AK, available phosphorous; SCAT, soil catalase; SUE, soil urease; SACP, soil acid phosphatase. In in each column different letters indicate significant different among the treatments at 0.05 level.

### Sugarcane Rhizosphere Microbial Diversity and Community Composition Under Different N Treatments

**FIGURE 2:**
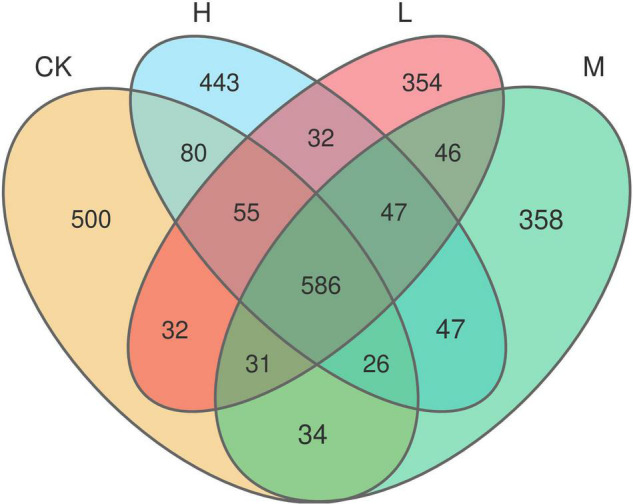
Venn diagram analysis representing the total and unique number of OTUs in each Treatment.

A total of 653,722 high quality reads of bacterial 16S rRNA were obtained from V5 to V7 region after screening, pre-clustering, and chimera removal with an average read of 54,476 ± 5889.50 read per sample (min = 43,744, max = 62,455). The details about each sample are depicted in Supplementary Figure 1. A total of 3,803 OTUs were obtained after filtering at minimum count of 4 and 20% prevalence in the samples, in which 586 OTUs were shared by all the samples (Figure 2). The number of unique OTUs in Ck, H, L, and M treatments were 500, 443, 354, and 358, respectively. In order to assess the rhizosphere microbial diversity subjected to different treatments, alpha diversity indices were studied (Table 2). The rarefaction curve illustrated enough richness of observed OTU and sequencing depth to examine microbial alpha diversity (Supplementary Figure 2). Different treatments significantly influenced the diversity indices, except Chao1 and ACE. Treatments M and H exhibited the highest Shannon, Chao1, ACE, and Simpson as compared to L and CK. Moreover, the highest alpha diversity was exhibited by treatment M, except Ace index, which suggested that nitrogen application could enhance the sugarcane rhizosphere bacterial community grown in fields. The medium N application, in particular, had the most impact.

**TABLE 2:** Sugarcane rhizosphere alpha diversity under different nitrogen application.

Treatments	Shannon	Chao1	ACE	Simpson
**CK**	6.85 ± 0.12b	1102.3 ± 53.3a	1135.2 ± 52.8a	0.973 ± 0.004b
**L**	7.07 ± 0.08*ab*	1120.8 ± 50.8a	1152.4 ± 54.8a	0.982 ± 0.001a
**M**	7.28 ± 0.05a	1197.5 ± 33.8a	1231.6 ± 31.1a	0.983 ± 0.001a
**H**	7.1 ± 0.14a	1158.1 ± 62.6a	1298.3 ± 48.7a	0.982 ± 0.001a
**F**	6.34	1.73	1.00	9.60
**P**	<0.05	= 0.25	= 0.45	<0.05

*Level of significance among each parameter is denoted by different letters in each column at 0.05 level.*

**FIGURE 3:**
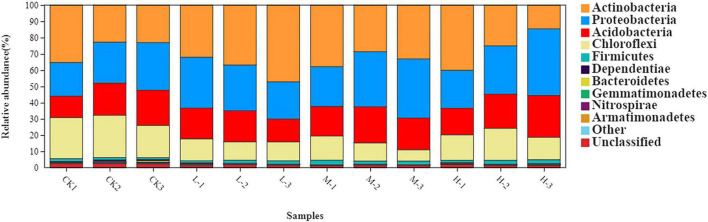
Relative abundance of major bacterial phyla under four different nitrogen application.

The rhizosphere soil bacterial communities in four different N applications were mainly dominated by Actinobacteria (31.94%), Proteobacteria (29.24%), Acidobacteria (19.52%), Chloroflexi (15.48%), and Firmicutes (1.85%) (Figure 3 and Supplementary Table 1). Moreover, the relative abundance of Acidobacteria, Actinobacteria, and Proteobacteria increased by 47, 61.6, and 71%, respectively, in M treatment as compared to CK. However, the relative abundance of Chloroflexi decreased by 39.4% in M treatment as compared to Ck. Results of this experiment demonstrated that nitrogen treatments affected soil bacterial community composition as well as the relative abundances of dominant bacterial phyla.

**FIGURE 4:**
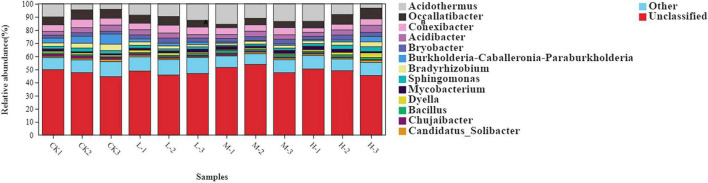
Relative abundance of major bacterial genus under four different nitrogen application.

At genus level the rhizosphere bacterial community under different nitrogen treatments mainly comprised of *Acidothermus* (9.28%), *Occallatibacter* (5.88%), *Conexibacter* (4.96%), *Acidibacter* (3.64%), *Bryobacter* (2.92%), *Burkholderia* (2.65%), *Bradyrhizobium* (2.57%), and *Sphingomonas* (2.05%) (Figure 4 and Supplementary Table 2). The relative abundance of Acidothermus and Bradyrhizobium increased by 77.2 and 30.3% in M treatment as compared to the CK; however, the relative abundance of other bacterial genera was found to be increased with the Low nitrogen treatment.

### Beta Diversity and Microbial Community Structure Under Different N Treatments

**FIGURE 5:**
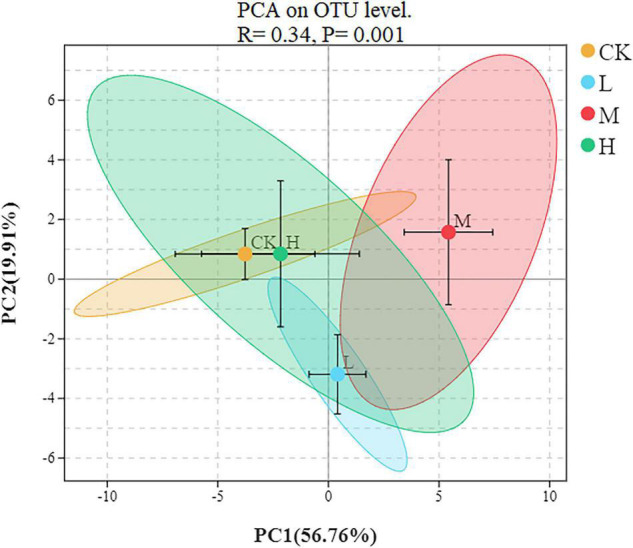
Principal component analysis at the OTU level of rhizosphere soil. Different ellipses indicate different treatment, the tick mark inside each ellipse represent standard error.

**FIGURE 6:**
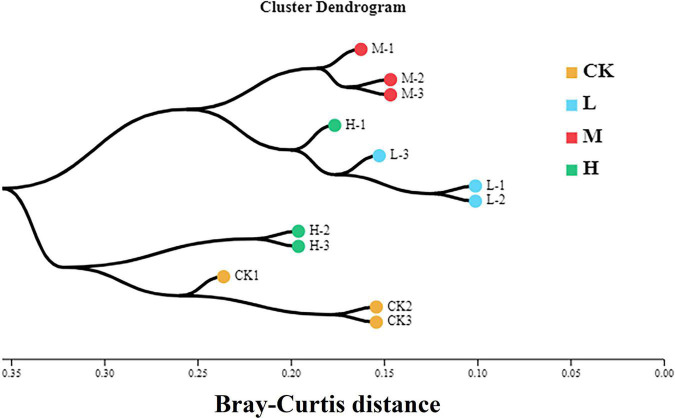
Hierarchical cluster analysis of the four different nitrogen treatments. Different color indicates samples from each treatment. The branching length of each sample indicates Bray-Curtis distance. Samples closer to 0 indicate similarity, while closer to 1 indicate dissimilarity.

To assess the bacterial beta diversity Principal component analysis (PCA) was carried out using Bray-Curtis algorithm (Figure 5). The plot explained 76.67% of the total variation between the treatments. Moreover, 56.76% of variation among the treatments was explained by PC1 while PC2 explained 19.91% of variation. Furthermore, the M treatment was distributed on the positive direction of PC1, while L nitrogen treatment was found primarily on the negative direction of PC1. The Ck and H treatments were distributed mainly in the positive direction of PC2. Meanwhile the M treatment was the only treatment positively correlated with the two quadrants of the principal components. The differences among the treatments were further confirmed by clustering analysis (Figure 6). According to the analysis, the rhizosphere soil samples clustered into two main groups, in which M nitrogen treatment clustered differently from the other treatments. The replicated samples from L treatment clustered together with one sample from H treatment, indicating similarity. The replicated samples from Ck treatment along with two samples from H treatment clustered into the second group indicating similarity between them.

### Network Correlation and Redundancy Analysis

**FIGURE 7:**
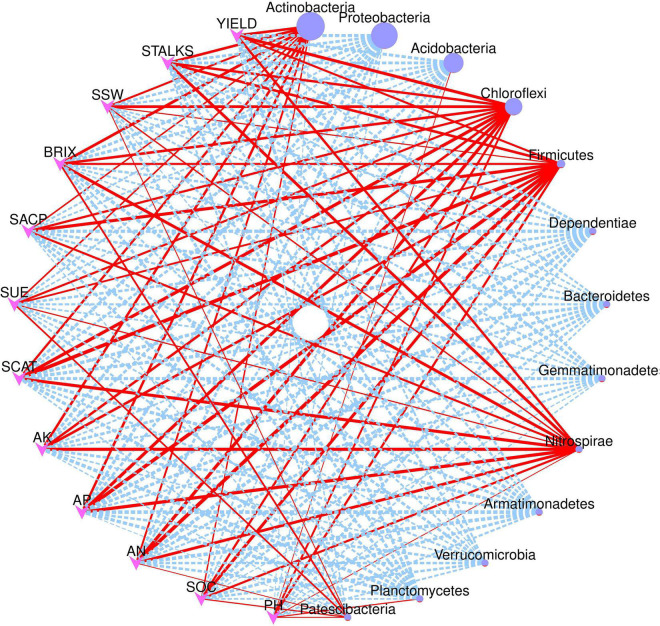
Network correlation analysis of environmental factors and yield parameters with major bacterial phyla. Red solid line indicates positive correlation while dotted blue lines indicate negative correlation. The size of the nodes represents abundance level of each phyla.

**FIGURE 8:**
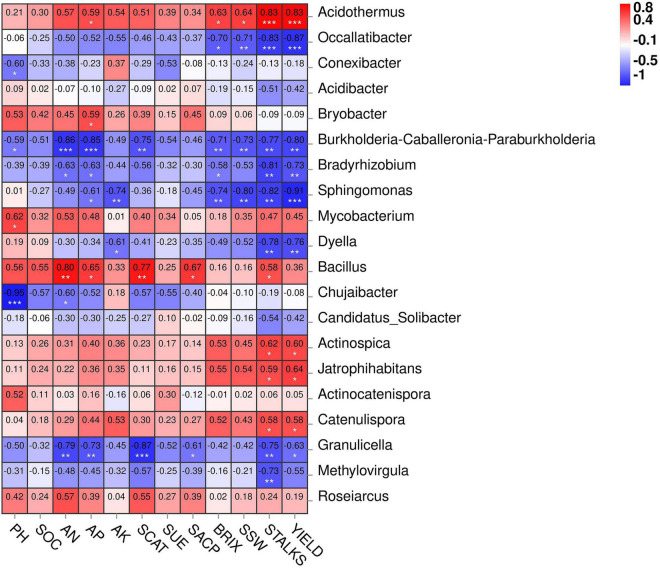
Heatmap of Spearman correlation between environmental factors and top 20 bacterial genera. The legend represents level of correlation. * Significance at *P* < 0.05, ** significance at *P* < 0.01, *** significance at *P* < 0.001.

Figure 7 depicts the network correlation among different environmental factors such as soil nutrients, pH, enzymatic activity, and sugarcane yield parameters with the top 10 abundant bacterial phyla in the rhizosphere soil subjected to different nitrogen fertilizer rates. Most of the studied parameters were found to be positively correlated with major bacterial phyla such as Actinobacteria, Chloroflexi, Firmicutes, and Nitrospirae. Proteobacteria was found to be negatively correlated with the studied parameters. Acidobacteria was positively correlated only with the soil pH. Moreover, the Spearman correlation among the environmental factors and top 20 bacterial genera are illustrated in Figure 8. According to the results, the genus *Acidothermus* has a significant positive correlation with yield parameters, while *Bacillus* has a significant positive correlation with soil environmental factors such as SACP, SCAT, AN, and AP. *Sphingomnas* and *Bradyrhizobium* showed a significant negative correlation with the environmental factors. Meanwhile, the Spearmen correlation analysis of environmental factors with top 20 abundant OTUs are depicted in Supplementary Figure 3.

**FIGURE 9:**
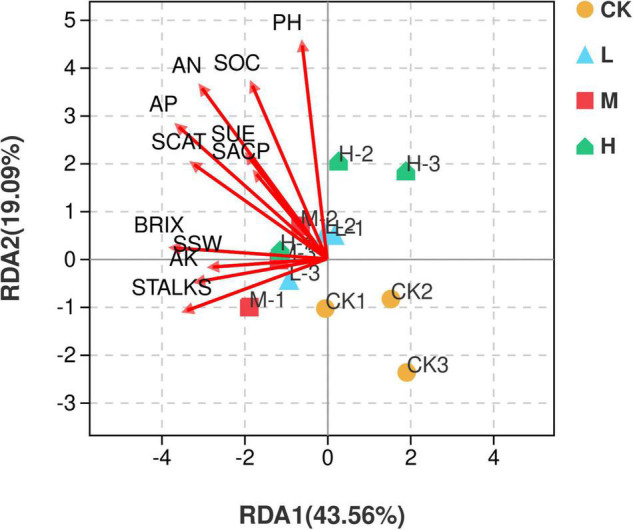
Distance-based Redundancy analysis of different treatments and environmental factors including soil properties and yield parameters. Red arrows represent environmental factors, and the arrow length represents the degree of influence on different samples.

Redundancy analysis (RDA) illustrated that the first two quadrants explained 62.65% variation among the soil samples with different nitrogen application rates and environmental factors, with RDA1 explaining 43.56% variation and RDA2 explaining 19.09% variation. M nitrogen treatment was found to be positively correlated with the environmental factors, while the control treatment was negatively correlated with all the environmental factors (Figure 9).

## Discussion

Fertilizer application is one of the most popular approaches in agricultural production for increasing crop yields. Despite the gradual improvement in nutrient use efficiency in China farming activities over the last decade, a massive amount of inorganic fertilizers such as nitrogen, phosphorous, and potassium are still applied to farmland to improve crop yields, resulting in a slew of serious ecological issues like soil organic matter loss, low soil fertility, nutrient inefficiency, and soil degradation. The Guangxi Zhang autonomous region accounts for more than 60% of China’s total sugarcane production. However, extensive use of chemical fertilizers is still a concern in the region (Yuchi et al., 2017). Our study mainly focused on finding an optimized and environmentally friendly amount of chemical fertilizers for the mentioned region. Soil enzymes are generally recognized as playing an essential role in decomposing organic matter in soil through their certain biochemical functions (Ellert et al., 1997). The enzymes can both catalyze microbial activities in soil and stabilize soil structure, decompose organic waste, form organic matter and cycle nutrients (Doran and Zeiss, 2000), as well as maintain soil ecological properties. To date, there is ample evidence that soil physicochemical factors such as SOC, TP, TN, AP, AN, and AK are enhanced by bacterial diversity; and at the same time, certain fertilizers can ameliorate soil acidification to some extent (Wang et al., 2018). Fertilizer application and rhizosphere microbial activities are often investigated in these research studies, but they still need to evaluate the optimized application rate of chemical fertilizers.

Our results suggested that cane yield, sucrose content, and soil pH varied significantly under different nitrogen treatments, which could be attributed to the fact that sugarcane root secretion or the rhizosphere microbiome under different levels of nitrogen recruits more functional microbes that aid in reducing soil acidity and promote uptake of important nutrient via roots. The sugarcane yield increases with different rates of nitrogen fertilizer, however, the cane yield is statistically non-significant (Yang et al., 2021). Soil enzymes mainly come from microbes and are important bioactive proteins involved in the cycling of nutrients and indicators of soil quality and fertility (Yang et al., 2018; Zheng et al., 2018). The activities of soil enzymes, including urease, catalase, and acid phosphatase in sugarcane field were altered under N application particularly with the application of Medium nitrogen fertilizer as compared to Ck (Table 1), however, SCAT was the only enzyme with significant differences. Higher soil enzyme activities can improve the N and P supply capacity of plant soil (Adetunji et al., 2017). SUE enzyme activity was found to be higher in M treatment; which could be attributed to the fact that urease activities in soil generally increased with urea fertilization to a certain limit. However, the high nitrogen rate in this experiment resulted in decreasing urease activity which is supported by the previous work (Sun et al., 2019), which reported a decrease in urease activities with an increase in nitrogen application rate. Catalase is an important redox enzyme for soil humus synthesis and prevents hydrogen peroxide toxicity by breaking it down into hydrogen and oxygen, while acid phosphatase enzyme provides phosphorous through hydrolysis (Stursova and Baldrian, 2011). The soil fertility and microbial activities can be effectively altered with external changes (Wang et al., 2012); therefore, enzyme activity is employed as a sensor to evaluate soil fertility status (Kaye et al., 2005; Acosta-Martinez et al., 2010). Our study found that the activities of the studied three enzymes was positively and significantly correlated with many important bacterial genera such as *Acidothermus* and *Bacillus*.

Chemical characterization is a well-known indicator for soil quality and microorganisms’ abundance determination (Fierer et al., 2007). Application of nitrogen fertilizers has been shown to benefit the accumulation of soil organic matter and consequently increase several elements of soil fertility and bacterial diversity (Sessitsch et al., 2001), as seen by the higher bacterial diversity in fertilizer impacted soil in our study. The higher amount of SOC in fertilizer impacted soil was associated with a high bacterial population, which could be explained in part by the fact that soil organic carbon is a good and reliable indicator of soil fertility. With an increase in the SOC of soil, it has been reported that activity of urease also increases (Adetunji et al., 2017). Agricultural practices such as use of nitrogen fertilizer can influence the bacterial population richness and diversity by altering the physiochemical features of the soil (Postma-Blaauw et al., 2010). Furthermore, the analysis of soil physiochemical properties also indicated that medium nitrogen application had significant differences in SOC, AP, AK, and AN (Table 1). Mineralization of organic compounds and biological nitrogen fixation are important activities performed by the microbial communities that provide available nitrogen to the plants (Schmidt et al., 2019).

Climate change, pesticides, and chemical fertilizer application are among major factors that might stymie natural selection in agroecosystem (Mhete et al., 2020). This has a distinct impact on the diversity and abundance of soil microbiota. Furthermore, the role of microorganisms is prominent in agricultural ecosystems, and with people becoming cognizant of the impact of nitrogen fertilizer on soil microbial population, the said work has become a topic of interest for researchers (Zhou et al., 2017; Wang et al., 2018; Yang et al., 2021). This research reflect that nitrogen fertilizers had an impact on the rhizosphere bacterial richness and evenness (Table 2). However, the most noticeable results were obtained through Medium fertilizer rate as compared to Low and High, which contrast with earlier studies showing decrease in soil bacterial biodiversity (Ling et al., 2017; Zhang et al., 2018). In this research the relative abundance of Actinobacteria and Proteobacteria increased significantly by the addition of nitrogen fertilizers as compared to CK (Supplementary Table 1), which are in line with the report of Yang et al. (2021). The species of Actinobacteria play a prominent role in the cycling of soil nutrients including carbon, phosphorous, potassium, nitrogen, and many others (Zhang et al., 2018) that produce sufficient water breaking enzymes which degrade lignin, chitin, and cellulose; however, its distribution is associated with environmental factors like soil acidity and type (Eisenlord and Zak, 2010; Duran et al., 2015). The second dominant phyla Proteobacteria is a group of Gram-negative bacteria that is involved in manipulation of soil nutrients (Spain et al., 2009). The presence of Proteobacteria in this study is in line with the literature, which reveals that presence of Proteobacteria is varied with level of nitrogen and crop selection (Gu et al., 2021). Furthermore, *alphaproteobacteria* was identified a major and abundant class in the rhizosphere samples of nitrogen fertilizers (Supplementary Figure 4). Species of this class such as *Sphingomonadales* and *Rhizobales* are reported to be involved in the degradation or breakdown of inorganic compounds nitrogen fixation (Liu and Liu, 2013). Based on all these results it can be concluded that optimum nitrogen fertilizer application is an effective way of manipulating soil microbial biodiversity and nutrients. As soil rhizosphere diversity is an important factor in reflecting soil fertility status; our results interpreted that Medium nitrogen application (150 kg ha^–1^) maximizes sugarcane yield and maintains soil health and bacterial diversity.

## Conclusion

In this study we investigated the composition of rhizosphere bacteria, soil physiochemical properties, and yield of sugarcane subjected to different nitrogen fertilizers rate in Guangxi. Significant changes in the said parameters through different nitrogen fertilizers were observed. The main cause of these changes could be attributed to the interaction of soil pH, rhizosphere microbes, and nutrients through fertilizer, however, the most integral enhancement was found through Medium (150 kg ha^–1^) nitrogen application. The Low and High nitrogen application altered the rhizosphere bacterial composition and sugarcane agronomic performance, however, the Medium application rate appeared to be significant, ecofriendly, and have an appropriate nitrogen rate for the region, exhibiting the utmost cane yield. Moreover, important bacterial phyla abundance and their composition was also found in the Medium nitrogen application rate. In light of all above results and interpretation, it is suggested that Medium (150 kg ha^–1^) nitrogen application rate is ecofriendly and productive and could be further used in the Guangxi region.

## Data Availability Statement

The datasets presented in this study can be found in online repositories. The names of the repository/repositories and accession number(s) can be found below: https://www.ncbi.nlm.nih.gov/, accession ID: PJRNA798064.

## Author Contributions

MZ and AK conceived the main idea of research. AK wrote the manuscript. MZ, HJ, and MAH revised the manuscript and provided suggestions. In addition, MA, JB, and AK analyzed the data. MA, JB, and SWG assessed in data collection. MZ supervised the work and provided financial support. All authors contributed intellectually to this study and assisted in manuscript preparation.

## Conflict of Interest

The authors declare that the research was conducted in the absence of any commercial or financial relationships that could be construed as a potential conflict of interest.

## Publisher’s Note

All claims expressed in this article are solely those of the authors and do not necessarily represent those of their affiliated organizations, or those of the publisher, the editors and the reviewers. Any product that may be evaluated in this article, or claim that may be made by its manufacturer, is not guaranteed or endorsed by the publisher.
